# Dendrimers Bind Antioxidant Polyphenols and cisPlatin Drug

**DOI:** 10.1371/journal.pone.0033102

**Published:** 2012-03-13

**Authors:** Amine Abderrezak, Philippe Bourassa, Jean-Sebastian Mandeville, Reza Sedaghat-Herati, Heidar-Ali Tajmir-Riahi

**Affiliations:** 1 Département de Chimie-Biologie, Université du Québec à Trois-Rivières, Trois-Rivières, Québec, Canada; 2 Department of Chemistry, Missouri State University, Springfield, Missouri, United States of America; Dalhousie University, Canada

## Abstract

Synthetic polymers of a specific shape and size play major role in drug delivery systems. Dendrimers are unique synthetic macromolecules of nanometer dimensions with a highly branched structure and globular shape with potential applications in gene and drug delivery. We examine the interaction of several dendrimers of different compositions mPEG-PAMAM (G3), mPEG-PAMAM (G4) and PAMAM (G4) with hydrophilic and hydrophobic drugs cisplatin, resveratrol, genistein and curcumin at physiological conditions. FTIR and UV-visible spectroscopic methods as well as molecular modeling were used to analyse drug binding mode, the binding constant and the effects of drug complexation on dendrimer stability and conformation. Structural analysis showed that cisplatin binds dendrimers in hydrophilic mode *via* Pt cation and polymer terminal NH_2_ groups, while curcumin, genistein and resveratrol are located mainly in the cavities binding through both hydrophobic and hydrophilic contacts. The overall binding constants of durg-dendrimers are ranging from 10^2^ M^−1^ to 10^3^ M^−1^. The affinity of dendrimer binding was PAMAM-G4>mPEG-PAMAM-G4>mPEG-PAMAM-G3, while the order of drug-polymer stability was curcumin>cisplatin>genistein>resveratrol. Molecular modeling showed larger stability for genisten-PAMAM-G4 (ΔG = −4.75 kcal/mol) than curcumin-PAMAM-G4 ((ΔG = −4.53 kcal/mol) and resveratrol-PAMAM-G4 ((ΔG = −4.39 kcal/mol). Dendrimers might act as carriers to transport hydrophobic and hydrophilic drugs.

## Introduction

**Figure 1:**
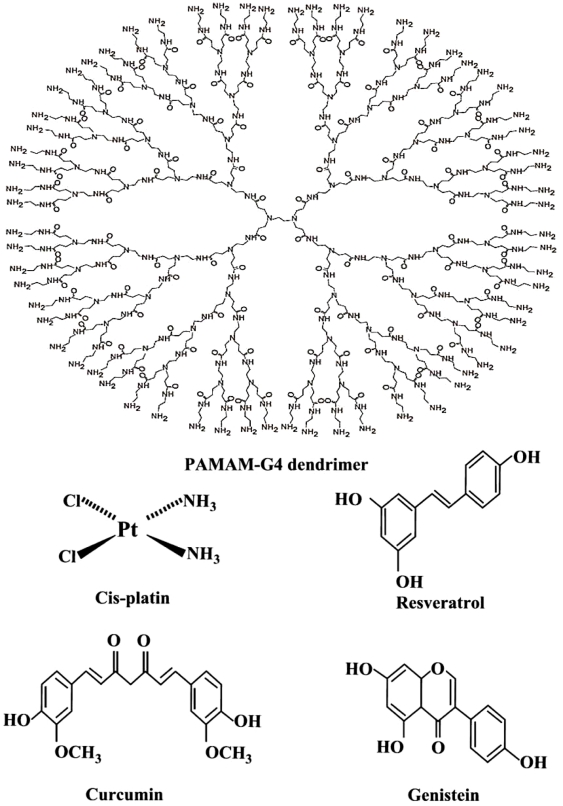
Chemical structures of PAMAM-G4 dendrimer, cisplatin, curcumin, resveratrol and genistein.

The generation of particular systems with a specific shape and size plays a crucial role in the development of modern drug delivery systems [1], [2]. Dendrimers are unique synthetic macromolecules of nanometer dimensions with a highly branched structure and globular shape [3], [4]. Among dendrimers, polyamidoamine (PAMAM) (Fig. 1) have received most attention as potential gene and drug delivery systems [5]–[8]. Several attempts have been made to design different dendrimers as drug carriers [9]. For example, anticancer fluorouracil drug was attached to the dendrimers with cyclic core [10] or using dendrimers having poly(ethylene glycol) grafts to encapsulate antitumor drugs adriamycin and methotrexate [11]. Similarly, it has been shown that a poly(propylene imine) dendrimer endcapped with 64 L-phenylalanine encapsulated nearly 4 molecules of Bengal Rose for every dendritic molecule [12]. Because dendrimers have a large number of terminal groups to which drug molecules can be attached, they can carry drug with a high efficiency. They contain several binding sites for hydrophobic, hydrophilic, cationic and anionic drugs. Using dendrimers for drug delivery, it is important to know their biological properties such as toxicity and biocompatibility. It has been demonstrated that, modification of the amino groups on the periphery of the dendrimer with poly(ethylene glycol) chains reduces the toxicity and increases the biocompatibility of the resulting polymer [13], [14]. This is because poly(ethylene glycol) is nontoxic, nonimmunogenic and water soluble, and its conjugation with other substrates, produces conjugates which combine the properties of both the substrate and the polymer. However, conjugate formation can alter the binding affinity of PAMAM to DNA or drug in general. In recent years, major attention has been focused on transporting drug by dendrimers [15], since these polymers contain several binding domains for different drugs complexation.

Curcumin (Cur) 1,7-bis-(4-hydroxy-3-methoxyphenyl)-1,6-heptadiens-3,5-dione (Fig. 1) the main yellow pigment of the powdered rhiome (turmeric) of the herb *curcuma longa* has been used for centuries as a spice and food coloring agent [16]. It has also been used to treat diseases such as inflammation, skin wounds and tumors as traditional medicine [17]. Curcumin exhibits antioxidant activity both *in vivo* and *in vitro* [17]. Apart from its anti-inflammatory, antimicrobial and antiviral properties curcumin is considered as cancer chemopreventive agent [18], [19].

Genistein (Gen) 4′,5,7-trihydroxy isoflavone (Fig. 1) presents in soybean and chick peas has a wide spectrum of physiological and pharmacological functions. It is known to antagonize human melanoma cell growth at G2/M transition [20] and found to inhibit H_2_O_2_/Cu(II) mediated DNA strand breaks acting as a direct scavenger of reactive oxygen species with the OH group at C-4 position responsible for its antioxidant activity [21].

Resveratrol (Res) 3,4′,5-trihydroxy stilbene (Fig. 1), is a polyphenolic compound present in variety of dietary plants like grape, berries and peanuts and has been identified as potential cardioprotective and chemopreventive agent against chemical carcinogens [22]. It is known to arrest cell cycle at the transition phase from S to G2/M in SW480 human colorectal cells [23]. It is similar structurally to genistein having OH group at C-4 position with major role in antioxidant activity [21], [24].

Cis-dichlrodiamineplatinum(II) (cisPt or cisplatin) (Fig. 1), is an anticancer drug, which exerts its antitumor activity by binding DNA *via* intrastrand cross-links to d(GpG)(dG = deoxyguanosine) and to d(ApG) (dA = deoxyadenosine), interfering with DNA replication and transcription and causing cell death [25], [26].

Our designed question was to determine if the dendrimers are capable of complexation with hydrophobic and hydrophilic drugs and to examine the binding of different drugs with dendrimers in aqueous solution in order to propose a mechanism of action by which dendrimres transport drugs *in vitro*.

In this report, we present spectroscopic and docking results on the interactions of different drugs curcumin, resveratrol and genistein (mainly hydrophobic) and cisplatin (hydrophilic) with several dendrimers of different compositions PAMAM (G4), m-PEG-PAMAM (G3) and m-PEG-PAMAM (G4) in aqueous solution at physiological conditions, using constant polymer concentration and various drugs contents. Structural information regarding drug hydrophobic and hydrophilic bindings and the effects of drug complexation on dendrimer stability and conformation in drug-polymer complexes is reported here.

## Materials and Methods

### Materials

Resveratrol, genistein, curcumin and cisplatin were purchased from Sigma Chemical Co and used as supplied. PAMAM-G4 (MW 14214 g/mol) was purchased from Aldrich Chemical Co and used as supplied. mPEG-PAMAM-G3 (MW 13423 g/mol) and mPEG-PAMAM-G4 (MW 19214 g/mol) (mPEG block has a molecular weight of 5000 g/mol) were synthesized according to published methods [9], [27]. Other chemicals were of reagent grade and used without further purification.

### Preparation of stock solutions

Solution of dendrimer 1 mM was prepared in distilled water and diluted to various concentrations in Tris-HCl. Solutions of curcumin, resveratrol and genistein (1 mM) were prepared in water/ethanol 50/50%), while cisplatin sample (1 mM) was prepared in hot water solution. The pH of stock solutions was kept at 7±0.2.

### FTIR spectroscopy

Infrared spectra were recorded on a FTIR spectrometer (Impact 420 model), equipped with deuterated triglycine sulphate (DTGS) detector and KBr beam splitter, using AgBr windows. Solution of drug was added dropwise to the dendrimer solution with constant stirring to ensure the formation of homogeneous solution and to reach the target drug concentrations of 0.125, 0.25, and 0.5 mM with a final dendrimer concentration of 0.5 mM. Spectra were collected after 2 h incubation of drug and polymer at room temperature, using hydrated films [28]. Interferograms were accumulated over the spectral range 4000-600 cm^−1^ with a nominal resolution of 4 cm^−1^ and 100 scans. The difference spectra [(drug+dendrimer solution) – (dendrimer solution)] were generated [28].

### UV-Visible spectroscopy

The UV-Vis spectra were recorded on a Perkin-Elmer Lambda spectrophotometer with a slit of 2 nm and scan speed of 400 nm min^−1^. Quartz cuvettes of 1 cm were used. The absorbance assessments were performed at pH 7.0 by keeping the concentration of dendrimer constant (0.14 mM), while varying the concentration of the pigment 0.025 mM to 0.0625 mM.

The binding constants were obtained according to the method described by Connors [29]. It is assumed that the interaction between the ligand L and the substrate S is 1∶1; for this reason a single complex SL (1∶1) is formed. It was also assumed that the sites (and all the binding sites) are independent and finally the Beer's law is followed by all species. A wavelength is selected at which the molar absorptivities ε_S_ (molar absorptivity of the substrate) and ε_11_ (molar absorptivity of the complex) are different. Then at total concentration S_t_ of the substrate, in the absence of ligand and the light path length is *b* = 1 cm, the solution absorbance is
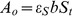
(1)In the presence of ligand at total concentration L_t_, the absorbance of a solution containing the same total substrate concentration is
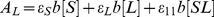
(2)(where [S] is the concentration of the uncomplexed substrate, [L] the concentration of the uncomplexed ligand and [SL] is the concentration of the complex) which, combined with the mass balance on S and L, gives
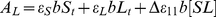
(3)where Δ*ε_11_ = ε_11_−ε_S_−ε_L_* (*ε_L_* molar absorptivity of the ligand). By measuring the solution absorbance against a reference containing ligand at the same total concentration L_t_, the measured absorbance becomes
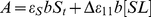
(4)Combining equation (4) with the stability constant definition K_11_ = [SL]/[S][L], gives
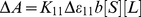
(5)where Δ*A = A−A_o_*. From the mass balance expression S_t_ = [S]+[SL] we get [S] = S_t_/(1+*K*_11_[L]), which is equation (5), giving equation (6) at the relationship between the observed absorbance change per centimeter and the system variables and parameters.
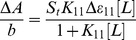
(6)Equation (6) is the binding isotherm, which shows the hyperbolic dependence on free ligand concentration.

The double-reciprocal form of plotting the rectangular hyperbola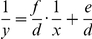, is based on the linearization of equation (6) according to the following equation,
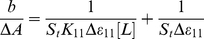
(7)Thus the double reciprocal plot of 1/ΔA versus 1/[L] is linear and the binding constant can be estimated from the following equation
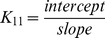
(8)


### Molecular modeling

The PAMAM-G4 and polyphenols structures were generated using the ChemOffice Ultra 6.0 software suite. The polyphenol were then automatically docked to the rough PAMAM-G4 structure using ArgusLab 4.0.1 (ArgusLab 4.0.1, Mark A. Thompson, Planaria SoftwareLLC, Seattle,WA, http://www.arguslab.com). The docked polyphenol-PAMAM-G4 structures were optimized by means of molecular dynamics using the MM+ force field available in HyperChem Pro 7.0. The heat time and run time for the simulations were 2 ps and 28 ps respectively with a step size of 0.001 ps. The temperature was initially set at 1 K and gradually increased to 300 K during the heat time by increments of 30 K. In all the simulations, equilibrium (achieving constant temperature near the selected final value) was reached after at most 20 ps. The free binding energies of the optimized PAMAM-G4. Polyphenol complex structures were calculated using the Ascore scoring function provided in the ArgusLab software.

## Results

### FTIR Spectral analysis of drug-dendrimers

**Figure 2:**
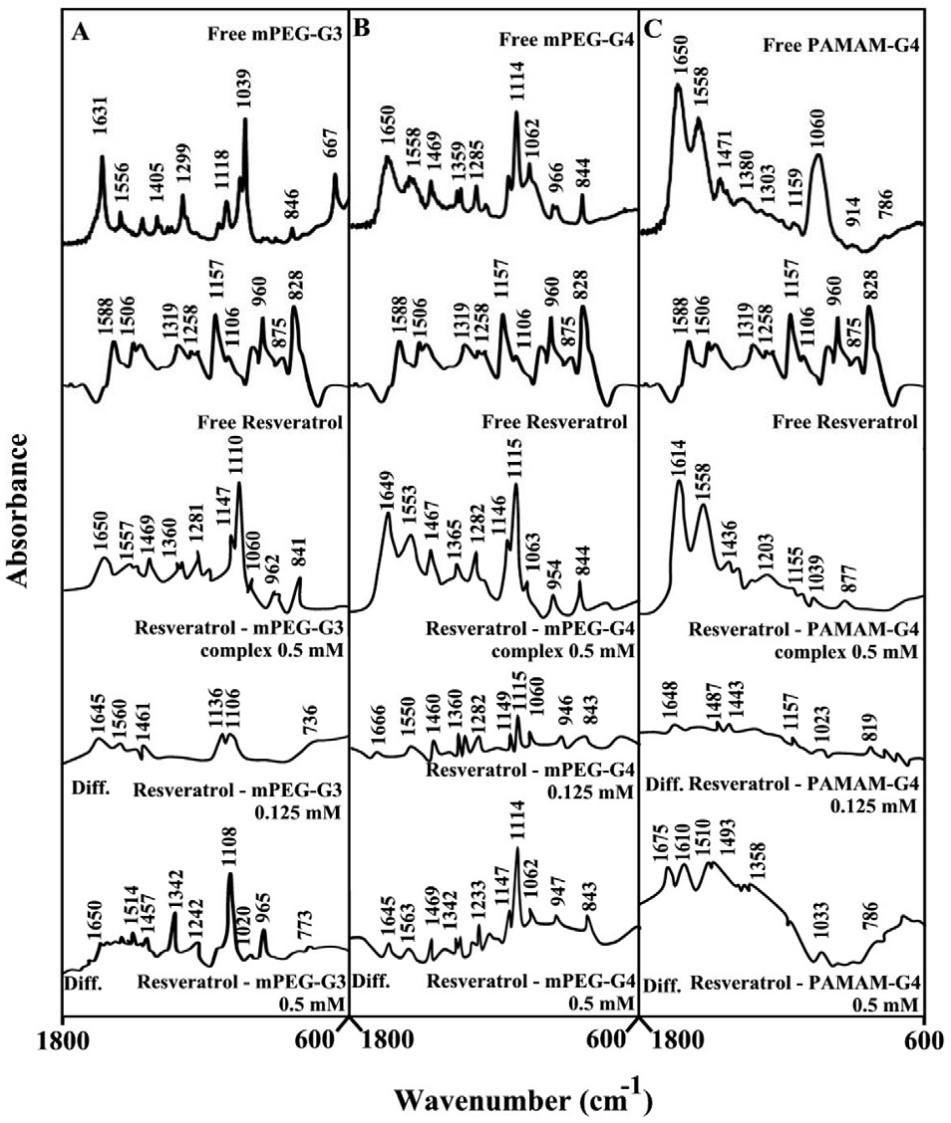
FTIR spectra and difference spectra (diff.) in the region of 1800-600 cm^−1^ of hydrated films (pH 7.4) for free mPEG-PAMAM-G3 (A), mPEG-PAMAM-G4 (B) PAMAM-G4 (C) (0.5 mM) and their resveratrol complexes obtained at different polyphenol concentrations (indicated on the figure).

**Figure 3:**
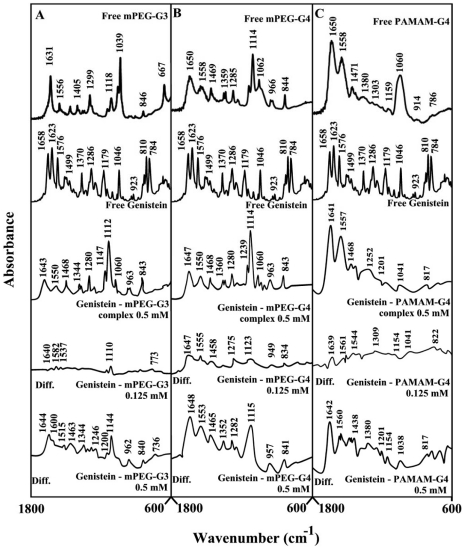
FTIR spectra and difference spectra (diff.) in the region of 1800-600 cm^−1^ of hydrated films (pH 7.4) for free mPEG-PAMAM-G3 (A), mPEG-PAMAM-G4 (B) PAMAM-G4 (C) (0.5 mM) and their genisteinl complexes obtained at different polyphenol concentrations (indicated on the figure).

**Figure 4:**
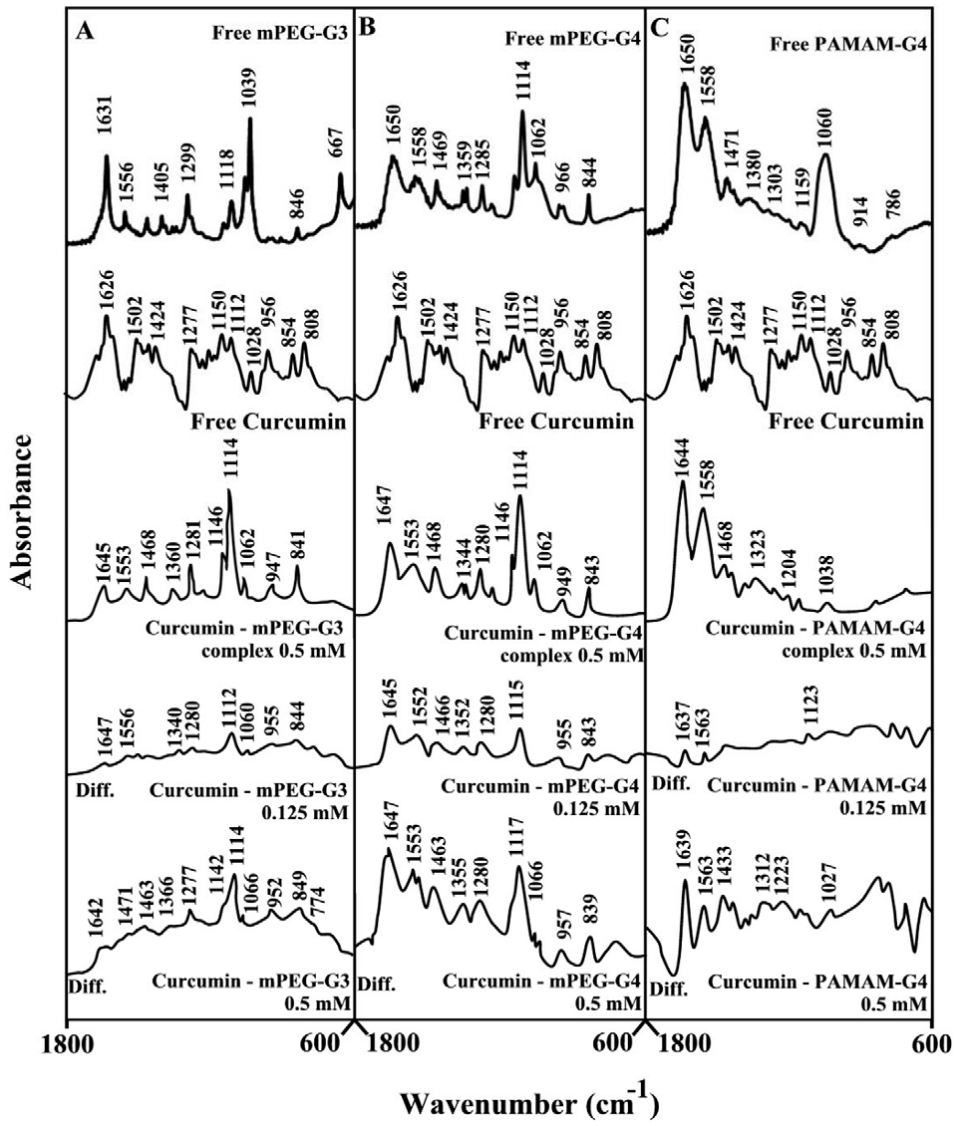
FTIR spectra and difference spectra (diff.) in the region of 1800-600 cm^−1^ of hydrated films (pH 7.4) for free mPEG-PAMAM-G3 (A), mPEG-PAMAM-G4 (B) PAMAM-G4 (C) (0.5 mM) and their curcumin complexes obtained at different polyphenol concentrations (indicated on the figure).

**Figure 5:**
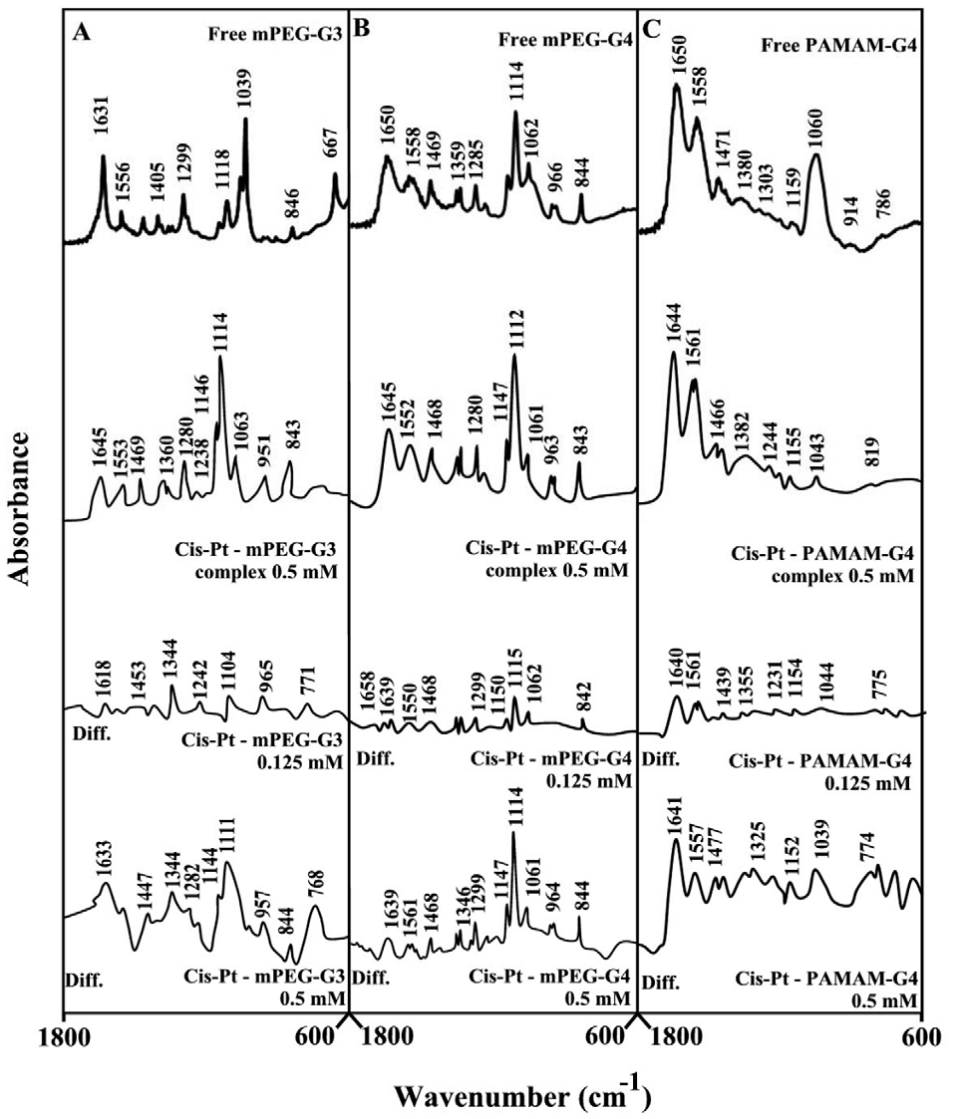
FTIR spectra and difference spectra (diff.) in the region of 1800-600 cm^−1^ of hydrated films (pH 7.4) for free mPEG-PAMAM-G3 (A), mPEG-PAMAM-G4 (B) PAMAM-G4 (C) (0.5 mM) and their cis-platin complexes obtained at different drug concentrations (indicated on the figure).

**Figure 6:**
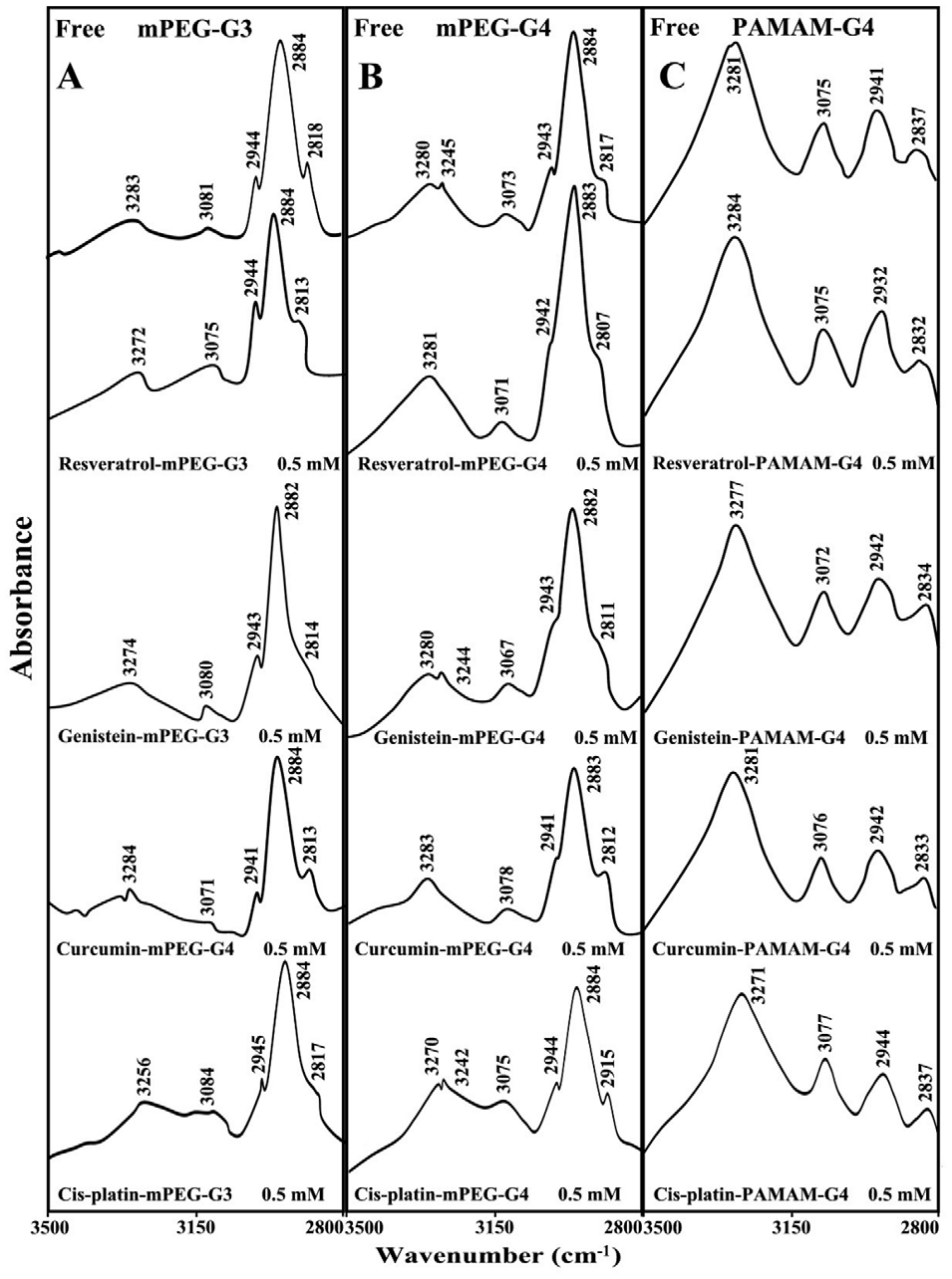
FTIR spectra in the region of 3300-2800 cm^−1^ of hydrated films (pH 7.4) for free mPEG-PAMAM-G3 (A), mPEG-PAMAM-G4 (B) and PAMAM-G4 (C) and their resveratrol. genistein, curcumin and cisplatin complexes obtained with 0.5 mM polymer and pigment concentrations.

The infrared spectral features regarding drug-dendrimer complexes are presented in Figures 2, 3, 4, 5, 6 and will be discussed for each drug-polymer separately.

In Figure 2, the infrared spectra and difference spectra of dendrimer complexes with resveratrol, are shown. Spectral shifting was observed for the polymer C = O, C-N, C-O stretching and NH bending modes [30], [31], due to drug hydrophilic interactions with polymer polar groups. The major infrared bands at 1631 (C = O stretch and NH bending), 1556 (C-N stretch), 1405, 1299 (C-O), 1118, 1061 and 1039 cm^−1^ (C-O and C-C stretch), in the infrared spectra of the free mPEG-PAMAM-G3 exhibited shifting and intensity increases in the spectra of resveratrol-mPEG-PAMAM-G3 complexes (Fig. 2A). Similarly, the major infrared bands of the free mPEG-PAMAM-G4 at 1650, 1558, 1469, 1359, 1285, 1114 and 1062 cm^−1^ showed shifting and intensity changes, upon resveratrol complexation (Fig. 2B). The infrared bands of the free PAMAM-G4 at 1650, 1558, 1471, 1380, 1159 and 1060 cm^−1^ were also shifted, upon resveratrol interaction (Fig. 2C). The observed spectral shifting was accompanied with gradual increase in intensity of the above vibrational frequencies in the difference spectra [(dendrimer+pigment solution) – (dendrimer solution)] of drug-polymer complexes (Fig. 2A, 2B and 2C, diffs). The spectral changes observed are attributed to the hydrophilic interactions of drug OH groups with dendrimer NH_2_, C-O and C-N groups. The hydrophilic interaction is more pronounced at high resveratrol concentrations as it evidenced by increase in the intensity of several positive features centered at 1650-1000 cm^−1^ in the different spectra of resveratrol-dendrimer complexes (Fig. 2A, 2B and 2C, compare diffs 0.125 and 0.5 mM).

Genistein-polymer complexation induced major spectral changes of the dendrimer infrared vibrational frequencies (Fig. 3). The spectral changes was observed mainly for the C = O, C-N, C-O stretching and NH bending modes [30], [31] in the region of 1650-1000 cm^−1^ of the infrared spectra of mPEG-PAMAM-G3, mPEG-PAMAM-G4 and PAMAM-G4, upon genistein complex formation (Fig. 3A, 3B and 3C). The spectral shifting was associated with increase of intensity of these vibrations observed in the difference spectra of genistein-dendrimer complexes (Fig. 3A, 3B and 3C, diffs). More perturbations of polymers spectra occurred at high genistein concentration (Fig. 3A, 3B and 3C, compare diffs of 0.125 mM and 0.50 mM). The observed spectral changes are attributed to the hydrophilic contacts of drug OH groups with dendrimer NH_2_, C-O and C-N groups.

Curcumin-dendrimer complexation caused more spectral changes than other polyphenol-polymer complexes (Fig. 4). Evidence for this comes from major shifting and intensity variations of the dendrimer in-plan vibrations (Fig. 4A, 4B and 4C). The major intensity increases occurred at high curcumin concentrations (Fig. 4A, 4B and 4C, compare diffs 0.125 mM and 0.50 mM). The observed spectral changes (shifting and intensity increases) are due to a major curcumin-polymer complexation via dendriemr C-O, C-N and NH_2_ groups with the polyphenol OH groups. The stronger curcumin-polymer interaction is due to the presence of several isomeric forms in solution (will be discussed furtheron), which enhances polyphenol-dendrimer complexation.

The infrared spectra of cis-platin-dendriemr complexes shown in Fig. 5 show cisplatin as a hydrophilic drug binds dendrimers *via* Pt cation and the polymer terminal NH_2_ groups. Evidence for this comes from major shifting of the polymer NH_2_ deformation mode at 1631 (mPEG-PAMAM-G3), 1650 (mPEG-PAMAM-G4) and 1650 cm^−1^ (PAMAM-G4) to 1645 (cis-Pt-mPEG-PAMAM-G3), 1645 (cisPt-mPEG-PAMAM-G4) and 1644 cm^−1^ (cisPt-PAMAM-G4) upon Pt complexation (Fig. 5A, 5B and 5C). The shifting of the NH_2_ bending mode was accompanied by a major shifting of the polymer NH stretching vibration at about 3280 cm^−1^ (will be discusses in hydrophobic interactions furtheron) upon Pt interaction.

### Hydrophobic interactions

The effect of drug-dendrimer complexation on the polymer antisymmetric and symmetric CH_2_ stretching vibrations in the region of 3000-2800 cm^−1^ was investigated by infrared spectroscopy [30], [31]. The antisymmetric and symmetric CH_2_ bands of the free mPEG-PAMAM-G3 located at 2944, 2884 and 2818 cm^−1^ were observed at 2944, 2984 and 2813 cm^−1^ (res-mPEG-PAMAM-G3) at 2943, 2982 and 2814 cm^−1^ (gen-mPEG-PAMAM-G3); at 2941, 2984 and 2813 cm^−1^ (cur-mPEG-PAMAM-G3); at 2943, 2984 and 2817 cm^−1^ (cisPt-mPEG-PAMAM-G3) (Fig. 6A). The antisymmetric and symmetric CH_2_ bands of the free mPEG-PAMAM-G4 located at 2943, 2884 and 2817 cm^−1^ were at 2942, 2983 and 2807 cm^−1^ (res-mPEG-PAMAM-G4); at 2943, 2982 and 2811 cm^−1^ (gen-mPEG-PAMAM-G4); at 2941, 2983 and 2812 cm^−1^ (cur-mPEG-PAMAM-G4); at 2944, 2984 and 2857 cm^−1^ (cisPt-mPEG-PAMAM-G4) (Fig. 6B). The CH_2_ stretching vibrations of the free PAMAM-G4, were at 2941 and 2837 cm^−1^ and shifted at 2932 and 2832 cm^−1^ (res-PAMAM-G4); at 2942 and 2934 cm^−1^ (gen-PAMAM-G4); at 2942 and 2833 cm^−1^ (cur-PAMAM-G4) and at 2944 and 2837 cm^−1^ (cisPt-PAMAM-G4) (Fig. 6C). The shifting of the dendrimer CH_2_ stretching vibrations was more pronounced for resveratrol, genistein and curcumin than for cis-platin-dendrimers (Fig. 6A, 6B and 6C). The observed spectral shifting for polymer CH_2_ vibrations is indicative of major hydrophobic interactions for resveratrol, genisten and curcumin with respect to cisPt-dendrimers. This is due to the major hydrophobic parts associated with pigment structure in comparison with inorganic cisplatin drug. However, cisplatin-polymer binding is mainly through hydrophilic interaction of Pt cation with dendrimer NH_2_ groups. Additional evidence for this come from major spectral shifting of the polymer NH stretching vibrations at 3283 (mPEG-PAMAM-G3), 3290 (mPEG-PAMAM-G4) and 3281 cm^−1^ (PAMAM-G4) towards a lower frequency at 3256 (cisPt-mPEG-PAMAM-G3), 3270 (cisPt-mPEG-PAMAM-G4) and 3271 cm^−1^ (cisPt-PAMAM-G4) (Fig. 6A, 6B and 6C). The larger shifting (Δυ = 27-10 cm^−1^) observed for polymer NH stretching vibration, is due to a major ionic interaction of polymer NH_2_ groups with Pt cation in comparison with the polyphenol-dendrimer complexes showing more hydrophobic interactions with minor perturbations of polymer NH stretching vibrations.

### UV-Visible spectra and stability of drug-dendrimers

**Figure 7:**
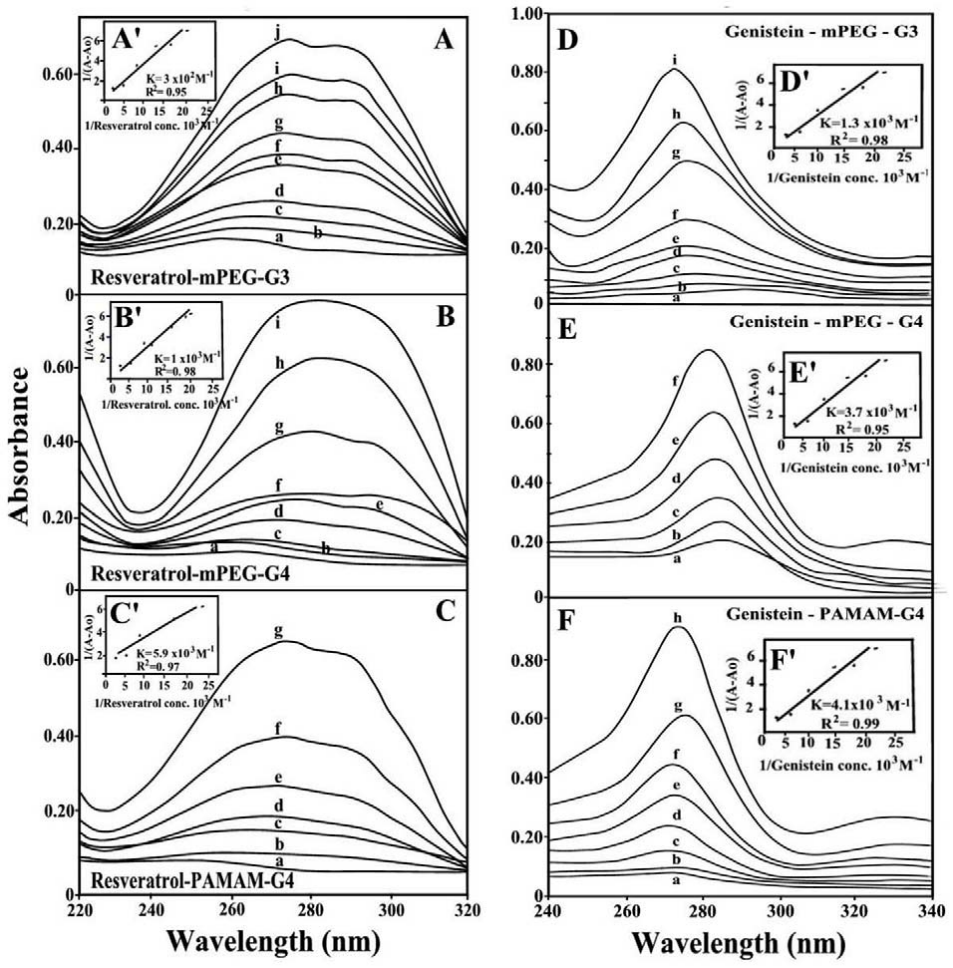
UV-visible spectra of mPEG-PAMAM-G3, mPEG-PAMAM-G4 and PAMAM-G4 and their complexes with resveratrol and genistein with free dendrimer at 100 mM and complexes b-I at 20 to 130 mM. (**A, B and C**) **Resveratrol with mPEG-PAMAM-G3, mPEG-PAMAM-G4 and PAMAM-G4 respectively.** (**D**, **E** and **F**) Genistein with mPEG-PAMAM-G3, mPEG-PAMAM-G4 and PAMAM-G4 respectively. (**A′**, **B′** and **C′**) Plots of 1/(A-A_0_) vs (1/pigment concentration) and binding constant (*K*) for Res-mPEG-PAMAM-G3, Res-mPEG-PAMA-G4, and Res-PAMAM-G4 respectively. (**D′**, **E′** and **F′**) Plots of 1/(A-A_0_) vs (1/pigment concentration) and binding constant (*K*) for Gen-mPEG-PAMAM-G3, Gen-mPEG-PAMA-G4, and Gen-PAMAM-G4 respectively.

**Figure 8:**
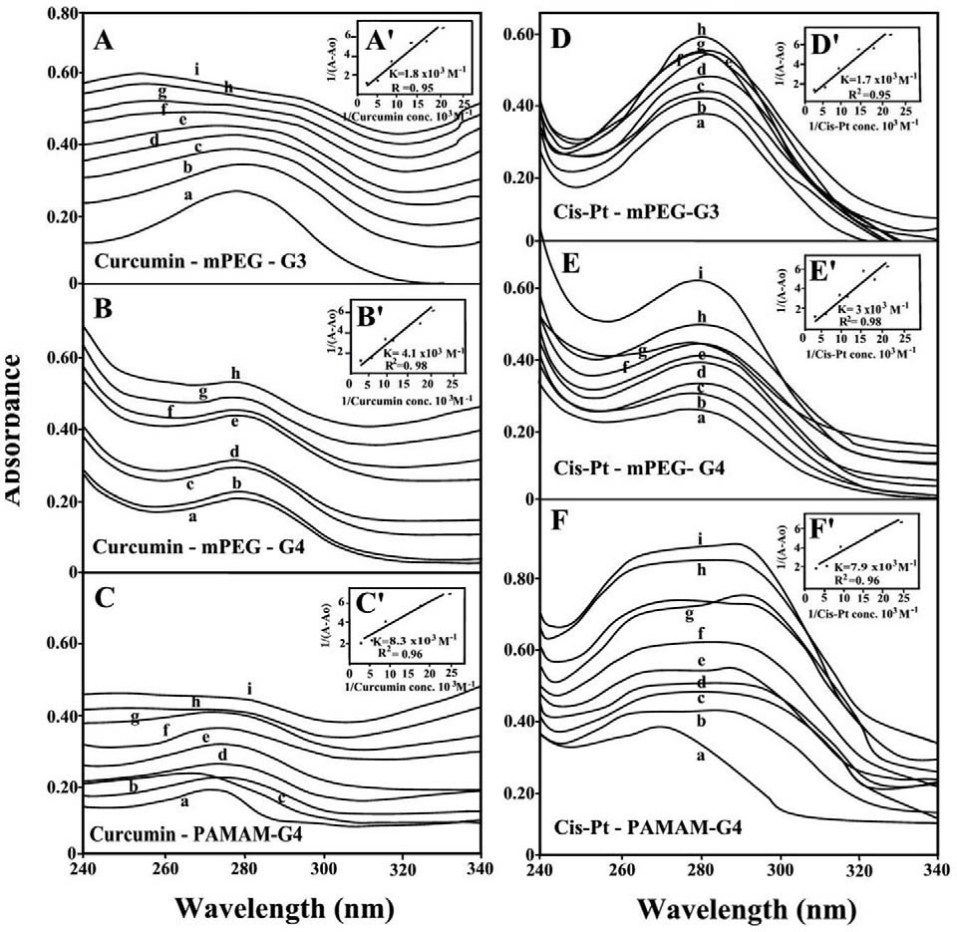
UV-visible spectra of mPEG-PAMAM-G3, mPEG-PAMAM-G4 and PAMAM-G4 and their complexes with curcumin and cisplatin with free dendrimer at 100 mM and complexes b-I at 20 to 130 mM. (**A, B and C**) **Curcumin with mPEG-PAMAM-G3, mPEG-PAMAM-G4 and PAMAM-G4 respectively.** (**D**, **E** and **F**). Cisplatin with mPEG-PAMAM-G3, mPEG-PAMAM-G4 and PAMAM-G4 respectively. (**A′**, **B′** and **C′**) Plots of 1/(A-A_0_) vs (1/pigment concentration) and binding constant (*K*) for Cur-mPEG-PAMAM-G3, Cur-mPEG-PAMA-G4, and Cur-PAMAM-G4 respectively. (**D′**, **E′** and **F′**) Plots of 1/(A-A_0_) vs (1/pigment concentration) and binding constant (*K*) for cis-mPEG-PAMAM-G3, cis-mPEG-PAMA-G4, and cis-PAMAM-G4 respectively.

The UV spectra of pigment-dendriemrs are presented in Figures 7 (resveratrol and genistein) and 8 (curcumin and cis-platin). It is clearly shown that as drug complexation occurs, major intensity increase of the dendrimer UV band centered at 260–290 nm was observed, due to polymer disaggregation and drug complex formation [32]. The spectral changes are more pronounced in the case of resveratrol, genistein, cisplatin than those of curcumin-dendrimers (Figs. 7 and 8). Curcumin with enolic form in solid state forms several isomeric species in solution (ketonic forms) with different affinities toward polymer complexation [33], [34]. Curcumin tends to form polymeric complexes with dendrimers and this can explain the differences in the UV spectral changes upon drug-polymer interactions (Figs. 7 and 8).

The drug-dendrimer binding constants obtained (according to the method described in experimental section) show one binding constant for each drug-polymer complex (Figs. 7 and 8 and Table 1). The affinity of dendrimer binding was PAMAM-G4>mPEG-PAMAM-G4>mPEG-PAMAM-G3, while the order of drug-polymer stability was curcumin>cisplatin>genistein>resveratrol (Table 1). The bigger affinity of PAMAM-G4 over mPEG-PAMAM-G3 and mPEG-PAMAM-G4 is due to the presence of 64 amino groups on the periphery of the dendrimer compared with 16 on mPEG-PAMAM-G4 and 8 on mPEG-PAMAM-G3 dendrimers.

**Table 1:** Calculated binding constants (*K*) for drug-dendrimer complexes.

DENDRIMERS	Curcumin (*K*)	Genistein (*K*)	Resveratrol (*K*)	Cis-platin (*K*)
**mPEG–G3**	**1.8** (±0.5)**×10^3^ M^−1^**	**1.3** (±0.3)**×10^3^ M^−1^**	**3.0** (±0.7)**×10^2^ M^−1^**	**1.7** (±0.3)**×10^3^ M^−1^**
**mPEG–G4**	**4.1** (±0.9)**×10^3^ M^−1^**	**3.7** (±0.6)**×10^3^ M^−1^**	**1.0** (±0.2)**×10^3^ M^−1^**	**3.0** (±0.7)**×10^3^ M^−1^**
**PAMAM-G4**	**8.3 (±0.9)×10^3^ M^−1^**	**4.1 (±0.6)×10^3^ M^−1^**	**5.9 (±0.7)×10^3^ M^−1^**	**7.9** (±0.8)**×10^3^ M^−1^**

### Docking study

**Figure 9:**
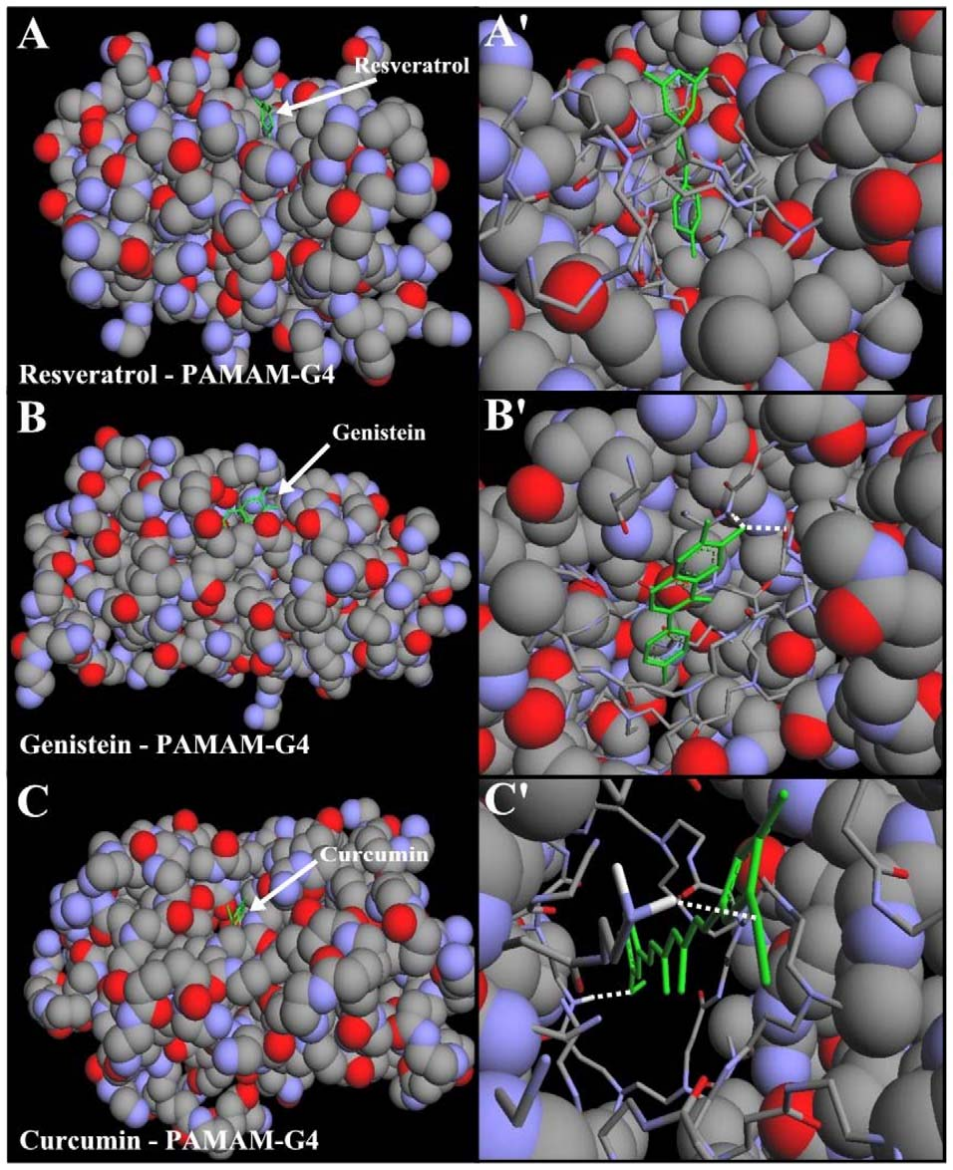
Optimized polyphenol-PAMAM-G4 docking structures. The polyphenols are shown in green color. (**A**) shows whole PAMAM-G4 in spheres with resveratrol and (**A′**) shows the zoom on the binding site represented in sticks. (**B**) shows whole PAMAM-G4 in spheres with genistein and (**B′**) shows the binding site represented in sticks. (**C**) whole PAMAM-G4 in spheres with curcumin and (**C′**) shows the binding site in represented in sticks.

Our results from FTIR and UV-visible spectroscopic methods were complemented with molecular dynamic simulations in which the resveratrol, genistein and curcumin were automatically docked to PAMAM-G4 and the resulting structure was optimized using the MM+ force field to determine the preferred conformations of the polyphenol-polymer complexes. The simulation results are shown in Fig. 9 and Table 2. The models showed that polyphenols are located in the cavities of PAMAM-G4 binding polymer in a hydrophobic fashion (Fig. 9). The free binding energy showed the order of binding genistein>curcumin>resveratrol (Table 2). This is due to a more hydrophobic nature of genistein in comparison with curcumin and resveratrol (Fig. 1). The dynamic process of drug-dendrimer binding is shown in 3 videos for curcumin-PAMAM-G4 (Video S1), for genistein-PAMAM-G4 (Video S2) and for resveratrol-PAMAM-G4 (Video S3).

**Table 2:** Binding energy for the best docking positions for drug-dendrimer Complexes.

Complex	Δ*G_binding_* (kcal/mol)
Resveratrol – PAMAM-G4	−4.39
Genistein – PAMAM-G4	−4.75
Curcumin – PAMAM-G4	−4.53

## Discussion

In constant search for designing tools to deliver drug *in vivo*, variety of synthetic polymers have been tested and used [35]. An ideal drug carrier must be biochemically inert and non-toxic, while protecting drug until it reaches the target molecule and releasing the drug. Among polymers, dendrimers are unique synthetic macromolecules of nanometer dimensions with highly branched structure and globular shape capable of transporting DNA, RNA and drug with high efficiency [4], [6]. These macromolecules have uniform size and are mono-dispersed with modifiable surface functionality as well as internal cavities. They contain several binding sites for hydrophobic, hydrophilic, cationic and anionic drugs (Fig. 1). Dendrimers can be used as a container which encapsulate drug molecules and carry to different targets *in vivo* [9], [36]–[38]. It has been shown that dendrimers with a hydrophobic interior and hydrophilic chain ends are able to solubilize hydrophobic compounds in aqueous solutions [38], [39]. Attempts have been made to design different dendrimers as drug carriers [9]. For example, anticancer fluorouracil drug was attached to the dendrimers with cyclic core [10] or using dendrimers having poly(ethylene glycol) grafts to encapsulate antitumor drugs adriamycin and methotrexate [11]. The complexation of dendrimers with anti-inflammatrory drug flurbiprofen was studied *in vitro* and *in vivo,* while drug biodistribution in different organs has been monitored [40]. Gene delivery targeted to brain has been attempted using transferring-conjugated polyethyleneglycol-modified polyamidoamine dendrimer [41]. The purpose of our investigation was to evaluate the potential of dendrimers as nanoscale drug delivery tools to carry hydrophilic (cisplatin) and hydrophobic drugs (resveratrol, genistein and curcumin). Resveratrol and genistein) with three OH groups and two and three phenolic rings, respectively are mainly hydrophobic in character and not soluble in water (Fig. 1). Curcumin in its keto form with two OH, two C = O and two methoxy groups also shows hydrophobic character, while soluble in ethanolic solution (Fig. 1). However, cisplatin drug is an example of hydrophilic drug soluble in water.

Infrared spectroscopic data in the region of 1700-1000 cm^−1^, where most of the polymer in-plane vibrations related to C = O, C-N, NH and C-O modes are located exhibited spectral changes (shifting and intensity variations) upon drug-polymer complexation. These changes were more profound in the case of cisplatin drug with respect to resveratrol, genistein and curcumin-dendrimer complexes. It showed clearly that the more hydrophilic drug induced more perturbations of polymer hydrophilic group vibrational frequencies with the order of spectral changes cisplatin>genistein>curcumin>resveratrol (Figs. 2, 3, 4, 5). This can be expected since cisplatin drug is the most hydrophilic with genistein being more hydrophilic than curcumin and resveratrol. However, analyses of the polymer CH_2_ stretching region (2900-2800 cm^−1^) exhibited shifting of the bands due to the polyphenol-polymer complexation (Fig. 6). The order of spectral shifting was resveratrol>genistein>curcumin>cispltin indicating little hydrophobic interaction for cisplatin-polymer complexes (Fig. 6). However, considerable shifting of the NH stretching vibration at about 3280 cm^−1^ toward a lower frequency was observed in the sperctra of cisplatin-dendrimers indicating a major hydrophilic interaction *via* Pt cation and the polymer terminal NH_2_ groups (Fig. 6). The order of hydrophilic interaction was cisplatin>genistein>curcumin>resveratrol.

The stability of drug-polymer complexes varies as dendrimer composition changes. PAMAM-G4 with 64 terminal amino groups forms more stable complexes than those of mPEG-PAMAM-G4 with 16 terminal NH_2_ groups and mPEG-PAMAM-G3 with 8 amino groups (Table 1). Drug with both hydrophilic and hydrophobic affinities such as curcumin forms stronger complexes than cisplatin, genistein and resveratrol with the order of stability curcumin>cisplatin>genistein>resveratrol (Table 1).

Molecular modeling showed polyphenol interaction with the hydrophobic parts of dendrimer with genistein-PAMAM more stable than resveratrol and curcumin-polymer complexes due to a more hydrophobic nature of genistein.

### Conclusion

In summary, based on our spectroscopic and docking results dendrimers are capable of binding to both hydrophilic and hydrophobic drugs using terminal NH_2_ groups and internal cavities. The low binding constants of pigment-polymers 10^2^ to 10^3^ M^−1^ indicate a weak complexation and an easy dissociation of drug molecule from dendrimer complexes, which is essential for suitable delivery systems. Hydrophobic drugs such as resveratrol, genistein and curcumin are mainly bound to internal cavities with some degree of hydrophilic contact *via* pigment OH groups, while hydrophilic cisplatin drug bind mainly *via* hydrophilic contacts with terminal NH_2_ groups. However, dendrimers with more internal cavities are effective carriers for hydrophobic drugs. This study clearly showed the applications of spectroscopic and docking techniques in structural analysis of dendrimer complexes with hydrophilic and hydrophobic drugs that are of major importance in designing and developing efficient tool in drug delivery systems.

## Supporting Information

Video S1 **The dynamic process of drug-dendrimer binding is shown in a video for curcumin-PAMAM-G4.**
(AVI)

Video S2 **The dynamic process of drug-dendrimer binding is shown in a video for genistein-PAMAM-G4.**
(AVI)

Video S3 **The dynamic process of drug-dendrimer binding is shown in a video for resveratrol-PAMAM-G4.**
(AVI)
